# Dual-Polarization Ambient Backscatter Communications and Signal Detection

**DOI:** 10.3390/s24010223

**Published:** 2023-12-30

**Authors:** Youze Yang, Sen Yan

**Affiliations:** School of Information and Communications Engineering, Xi’an Jiaotong University, Xi’an 710049, China

**Keywords:** IoT sensors, ambient backscatter communications, dual-polarization, symbol detection

## Abstract

Ambient backscatter communication (AmBC), an emerging mechanism for batteryless communications that can utilize ambient radio-frequency signals to modulate information and thus reduce power consumption, has attracted considerable attention and has been considered as a critical technology in green “Internet of Things” sensor networks due to its ultra-low power consumption. This paper presents the first a complete dual-polarization AmBC (DPAm) system model, which can extend AmBC into polarization diversity and improve the data-transmission rate of backscatter symbols. We proposed two scenarios: direct dual-polarization-based DPAm node structures and polarization-conversion-based DPAm node structures. In addition, we consider a parallel backscatter mode with differential coding and develop corresponding detectors, which also give the analytical detection thresholds. Moreover, we consider a simultaneous backscatter mode with Manchester coding in order to avoid complex-parameter estimation. To address the power imbalance problem of the DPAm system that arises because the polarization deflection angle would cause the power level to change with different polarization patterns, we also develop a power-average detector and a clustering detector. Simulation results show the throughput performance on each DPAm node structure with each detector, demonstrating the feasibility and efficiency of the proposed DPAm nodes and detectors. Compared with single-polarization AmBC (SPAm), the proposed DPAm node can achieve higher throughput in most cases. Finally, the clustering detector is shown to be more robust to short training sequences and complex environments.

## 1. Introduction

Internet of Things (IoT) is a rapidly growing paradigm that involves things or objects interacting and has received widespread attention from both industry and academia [1,2]. IoT has many potential applications in the health-care, environmental, industrial, commercial, and smart-city fields that will profoundly change how humans live [3]. For the widely discussed 5th generation (5G) communication system, in recent years, one of the dominant communication paradigms has been the connection of a vast number of devices [4,5]. However, for such large-scale networks, nonnegligible issues include the power consumption, frequency spectrum crowding, and maintenance costs. For instance, traditional radio-frequency identification (RFID) readers require the active emission of an electromagnetic wave on specific frequency bands in order to identify RFID tags, though the RFID tags can use passive or ultralow power dissipation [2]. These costs restrict further device deployment in massive IoT sensor networks.

In the development of green IoT networks, AmBC has been considered to have potential as a technology for batteryless communications [6,7]. The authors in [8] first proposed the concept of AmBC systems, which allow backscatter devices utilize existing, ambient RF signals in their environments to communicate with each other or harvest energy to keep themselves working. The authors in [8] also designed AmBC prototypes to demonstrate the feasibility of employing AmBC systems that use ambient RF signals from TV towers. Applications such as smart-card systems and item detection in grocery stores have also been demonstrated to reveal the potential value of AmBC in practical uses of IoT sensor networks. Subsequently, numerous studies have developed AmBC systems that use different frequency bands and different forms of ambient RF signals as carriers, including frequency modulation (FM) radio [9,10], Bluetooth [11,12], and Wi-Fi APs [13,14]. AmBC systems have many advantages compared to traditional wireless communications and backscatter communications, i.e., (1) because they modulate information bits on ambient RF signals, AmBC systems do not require active components like oscillators and thus could save energy and circuit-design space; (2) because they harvest energy from the environment, AmBC systems would reduce dependence on large-capacity batteries and routine maintenance; (3) because they remove the specific-frequency-band readers, AmBC systems will reduce deployment and maintenance costs and avoid frequency spectrum crowding. Some researchers have also asserted that utilizing AmBC in wireless communication systems could improve outage capacity [15] and energy efficiency [16].

In order to improve the performance of AmBC systems, many novel designs have been proposed in recent years that aim to maximize the use of communication resources. The authors in [17] extended AmBC systems into dual-band designs and designed a dual-band AmBC system in order to allow AmBC devices to simultaneously utilize two ambient RF bands to achieve 4-QAM modulation. The authors in [18] designed an all-digital AmBC system by utilizing the impedance variation of the microcontroller IO pin to modulate information bits, further reducing the complexity and costs of AmBC systems. In [19,20], the high-order modulations M-FSK and M-PSK are implemented in AmBC systems to improve the data rate and get better symbol error rate performance than is achieved in conventional on–off keying (OOK) modulation. The authors in [21] incorporated spatial modulation into AmBC systems and significantly improved the throughput compared to that of a single-antenna AmBC by merging the index information of the active antenna into symbol backscatter.

However, it should be noted that few studies have focused on exploiting polarization patterns in AmBC systems, and most studies of AmBC systems have neglected the effect of polarization mismatch. Related works [22,23] provided preliminary experiments on polarization diversity in AmBC systems. In [22], the authors used rotation dipoles and reconfigurable antennas to modulate information by switching among polarization patterns. The results of simulations and experiments show that reconfigurable tags have better performance than non-reconfigurable tags. In [23], the authors designed a polarization conversion-based AmBC system that converts a linearly polarized ambient RF signal into a circularly polarized modulated signal in order to cancel the direct-path interference in AmBC systems. By deploying a reader with both left-handed and right-handed circularly polarized antennas and outputting the difference, the system cancels the linearly polarized ambient RF signal and detects the circularly polarized modulated signal. However, the experiments in these works consider only the ideal case wherein a single-frequency carrier is generated as an ambient RF signal in an anechoic chamber.

Moreover, channel estimation in AmBC systems is extremely difficult. Because the AmBC devices are ultra-low power devices that cannot operate channel-estimation algorithms, the channel coefficient of the path between the ambient RF source and the AmBC devices is unknown to the reader. This limitation presents many challenges for symbol detection in AmBC systems wherein the detection methods used in conventional wireless communications may not work. Several works that have made contributions regarding how to indirectly obtain or avoid channel estimation in AmBC systems [24,25,26,27]. In [24], the authors utilized differential coding in AmBC systems and proposed noncoherent detection methods, including a joint-energy detector, threshold derivation, and parameter estimation. In [25], the authors further proposed semi-coherent detection methods for u se in AmBC systems, in which several training symbols are used to estimate detection parameters. In [26], the authors utilized Manchester coding in AmBC systems and proposed the use of energy detectors. Each of the original symbols is implicitly contained in the relative values of the signal energy of the first and second halves of the Manchester-encoded symbols. This method eliminates the need to estimate the detection threshold. In [27], the authors examined the use of multi-antenna AmBC devices and proposed a detection method based on Bartlett’s test. The advantage of this approach is that it does not require knowledge of certain parameters, including the power of the ambient RF source and the channel coefficient. In addition to these conventional methods, machine learning has potential as a useful technology and has been used in several studies of AmBC signal detection in recent years [28,29,30,31,32]. Machine learning-assisted detection methods offer several advantages, such as the ability to find the intrinsic relationships within datasets and the ability to adapt autonomously to complex environments. These advantages would further reduce the need for parameter estimation and improve detection accuracy in AmBC systems.

Motivated by these studies, in this paper, we provide a study of a DPAm system that takes into account details of signal-propagation conditions, including the effect of polarization mismatch. We present two different structures for backscatter nodes along with their corresponding transmission models for a DPAm system. Then, we explore a parallel backscatter mode that employs differential coding for each polarization pattern. This approach includes deriving the detection threshold in order to improve the symbol rate compared to that achieved in a SPAm system. Analytical throughput is also obtained. Moreover, we note that the variance in the ambient RF signal is greater than the variance in the channel coefficient, especially in the case of a high signal-to-noise ratio (SNR), which means the effect of the ambient RF signal is stronger. Hence, we also consider a simultaneous backscatter mode with Manchester coding on both polarization patterns. To deal with the problem of power imbalance caused by the randomness of the channel coefficients and polarization deflection angles, we propose a power average detector and a clustering detector for use in simultaneous backscatter mode in a DPAm system. The results of this work indicate that such a DPAm system could have significantly improved throughput compared to a SPAm system. In addition, the proposed clustering detector is more robust than the power average detector in short training sequences and complex environments.

Adopting polarization diversity in AmBC systems has obvious advantages. First and foremost, because the ambient RF signals are usually degraded by from severe reflection and backscattering caused by objects in the physical environment, e.g., buildings, trees, ground, rain and snow, they are already distorted and rotated by the time they reach the AmBC devices. Both a multipath effect and polarization mismatch result. Thus, the single-polarized antenna in the SPAm system will lose a portion of the ambient RF signal power for ambient backscatter communication due to the polarization mismatch, which will lead to a decrease in communication performance. Through the use of a dual-polarized antenna, the DPAm system has the ability to more fully exploit the ambient RF signals, especially for the extreme case wherein the arriving ambient RF signals are almost orthogonal to the single-polarized antenna. In such a case, the SPAm system will struggle to transmit information, but the DPAm system will maintain transmission on the other polarization pattern, showing the robustness of the system. In addition, the dual-polarized antenna in the DPAm system could backscatter the ambient RF signals into orthogonal channels, which will avoid the mutual interference of the massive-input-massive-output (MIMO) channels. Last but not least, dual-polarized antenna with elaborate designs could be smaller in size, which would reduce the physical size of the AmBC devices compared to array antennas. In summary, the proposed DPAm system has potential applications in dynamically complex environments, in particular wireless wearable sensors, object localization, and environmental monitoring [6,33,34].

The remainder of the paper is organized as follows. In Section 2, the DPAm node structures and transmission model are presented in detail. Section 3 describes the parallel backscatter mode with differential coding and the corresponding detector in the DPAm system. Section 4 describes the simultaneous backscatter mode with Manchester coding and corresponding detectors in the DPAm system. Numerical simulation results and discussions are presented in Section 5, and Section 6 concludes the paper.

## 2. System Model

### 2.1. Overview of DPAm System Model

**Figure 1:**
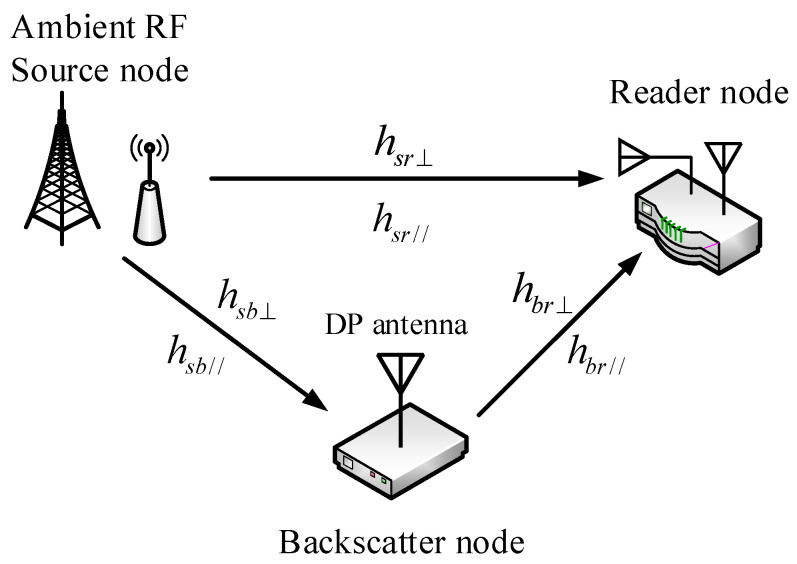
A model of an elementary bistatic AmBC system.

As shown in Figure 1, we consider an elementary bistatic AmBC system model consisting of an ambient RF source node, a backscatter node, and a reader node. In common situations, the ambient RF source node, e.g., TV tower, cellular BS, and Wi-Fi AP, has a vertical linear-polarized antenna. The reader node has two orthogonal polarization antennas, which can be linearly polarized [22] or circularly polarized [23]. As the reader antennas are very close to each other with respect to the distance between the ambient RF source node and the reader node, they can be approximately considered to be at the same location. Depending on the orthogonality of the received electromagnetic waves, different information bits can be modulated on different polarization patterns in order to improve the data rate of the AmBC system. Therefore, a dual-polarized antenna is essential for the backscatter node.

**Figure 2:**
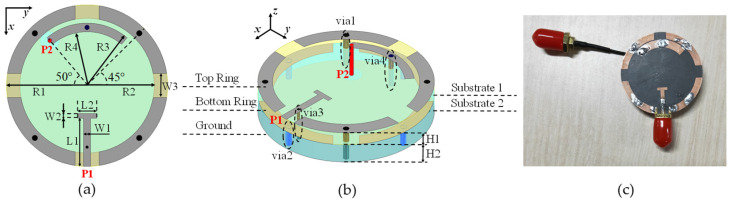
A dual-polarized smartwatch antenna [35]. Port 1 and Port 2 give orthogonal linear-polarization patterns: (**a**) top view, (**b**) 3-D view and, (**c**) fabricated antenna.

To fully explain our proposed system model and its potential applications, Figure 2 demonstrates a dual-polarized smartwatch antenna designed by our lab [35], which is suitable for wearable sensing and is capable of object localization and environment monitoring. This antenna has a cylindrical shape with right-/left-handed transmission line (CRLH-TL) metamaterial structure. There are three layers of metal parts and two layers of F4BM board substrates. The feeding structure of P1 is a coplanar waveguide (CPW) port with a T-shaped transmission line, while P2 is a coplanar strip (CPS) port with the annular patch-feeding transmission line. The antenna geometric parameters are also given in Table 1.

**Figure 3:**
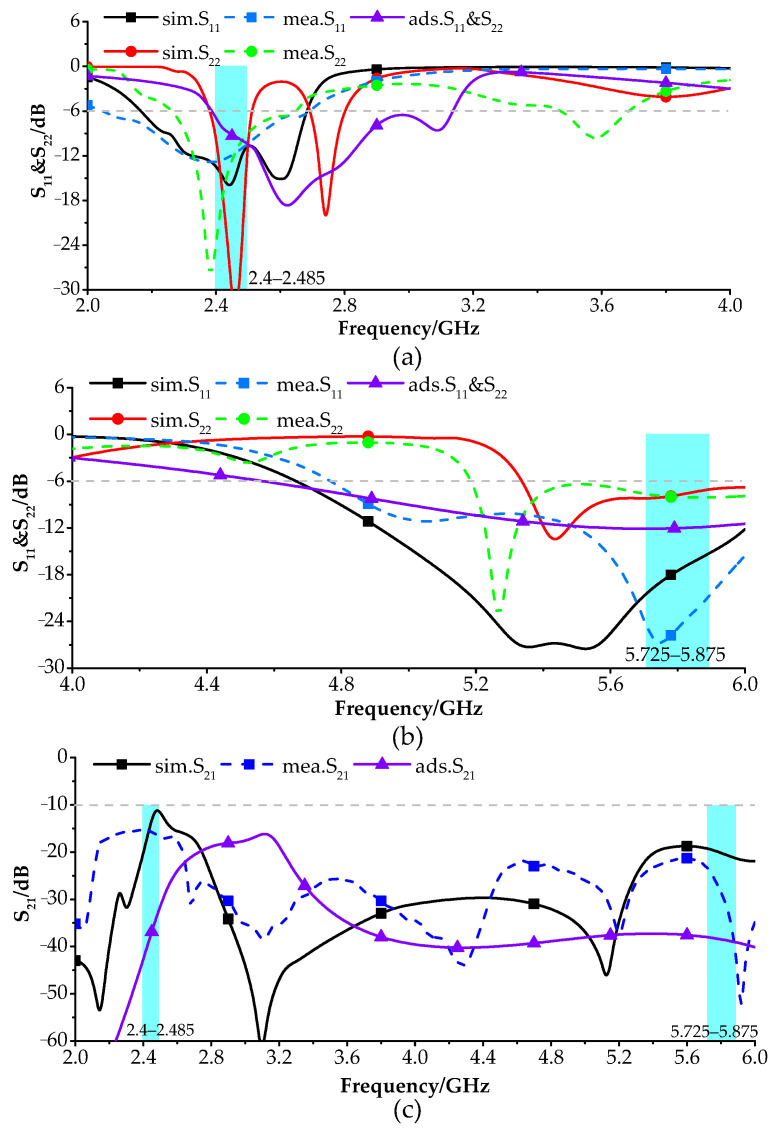
S-parameters in free space [35]: (**a**) S_11_ and S_22_ in 2–4 GHz band, (**b**) S_11_ and S_22_ in 4–6 GHz band and (**c**) S_21_ in 2–6 GHz band.

Figure 3 shows the simulated and measured S-parameters for an antenna in free space. For Port 1, the simulation shows an operating frequency range of 2.15 to 2.65 GHz and 4.63 to 6.15 GHz, while the actual measurements indicate a range of 2.05 to 2.65 GHz and 4.78 to 6.27 GHz. These ranges satisfy the criteria of S_11_ < 6 dB and VSWR < 3. For Port 2, the simulation shows an operating frequency range of 2.39 to 2.5 GHz and 5.32 to 6.11, while the actual measurements indicate a range from 2.26 to 2.64 GHz and 5.18 to 6.21 GHz These ranges satisfy the criteria of S_22_ < 6 dB and VSWR < 3. It can be readily seen that this antenna can operate in the 2.4/5.8 GHz WLAN band (2.4–2.485 and 5.725–5.875 GHz). The coupling parameters (S_21_) are also below −12 dB in both operating-frequency bands in each case, indicating excellent isolation.

**Figure 4:**
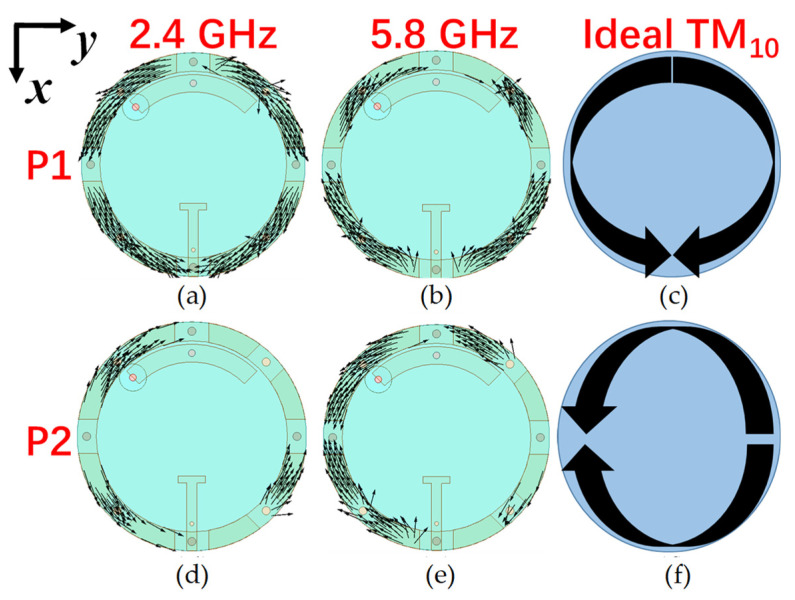
Current distributions [35]: (**a**) 2.4 GHz when P1 is activated. (**b**) 5.8 GHz when P1 is activated. (**c**) Ideal x-polarized TM 10 mode for annular rings. (**d**) 2.4 GHz when P2 is activated. (**e**) 5.8 GHz when P2 is activated. (**f**) Ideal y-polarized TM 10 mode for annular rings.

Figure 4 shows the current distributions at 2.4 and 5.8 GHz when either P1 or P2 is activated. When P1 is active with P2 connected to a matching load, the currents flow symmetrically from across the *x*-axis along the annular patch at 2.4 and 5.8 GHz, as shown in Figure 4a,b. This pattern is almost the same as the ideal *x*-polarized TM_10_ mode shown in Figure 4c. Also, when P2 is active and P1 is connected to a matching load, the current distributions flow symmetrically along the *y*-axis on the annular patch at 2.4 and 5.8 GHz, as shown in Figure 4d,e. This pattern is almost the same as the ideal *y*-polarized TM_10_ mode shown in Figure 4f. Although the currents flow in opposite directions when each port is activated at different frequency bands, they could achieve orthogonal linear polarization patterns at the same frequency band.

In our early experiments, this structure with dual-polarized antenna can backscatter electromagnetic waves independently and simultaneously on different antenna polarization patterns. We consider two structures of the backscatter node in the DPAm system.

### 2.2. Direct Dual-Polarized Backscatter (DDB)

In a typical office or home environment, the distance between the backscatter node (e.g., a smartwatch with antenna, as shown in Figure 2) and the reader node (e.g., a smartphone) is within 1 m, while the distance between the ambient RF source node (e.g., a Wi-Fi AP) and the backscatter node or the reader node is over 10 m. Hence, it is easy for the backscatter node to backscatter the ambient RF signal directly to the reader node from the existing ambient RF signal. We propose a simple DDB backscatter-node structure for these scenarios.

**Figure 5:**
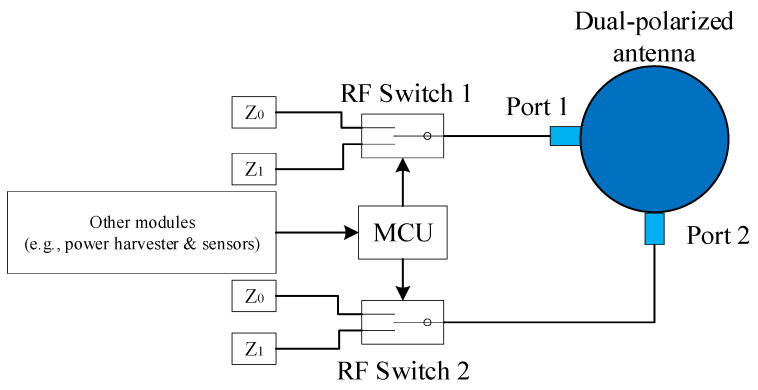
Backscatter node with DDB structure.

In this scenario, the backscatter node has only one dual-polarized antenna. For simplicity, we abstract the dual-polarized antenna shown in Figure 2 as a circle with two ports in the following figures. Two ports of the antenna connect to an RF switch separately. Each RF switch chooses either matched or mismatched impedance to modulate the backscatter signal with information. When the matched impedance is chosen, most of the signal of the corresponding polarization pattern will be absorbed, resulting in very little reflection. This scenario corresponds to a binary ‘0’. When mismatched impedance is chosen, most of the received signal of the corresponding polarization pattern is reflected. This scenario corresponds to a binary ‘1’. The control signal is given by a low-power microcontroller (e.g., MSP430 [8]), which modulates information on the backscatter signal. The DDB node structure is shown in Figure 5.

**Figure 6:**
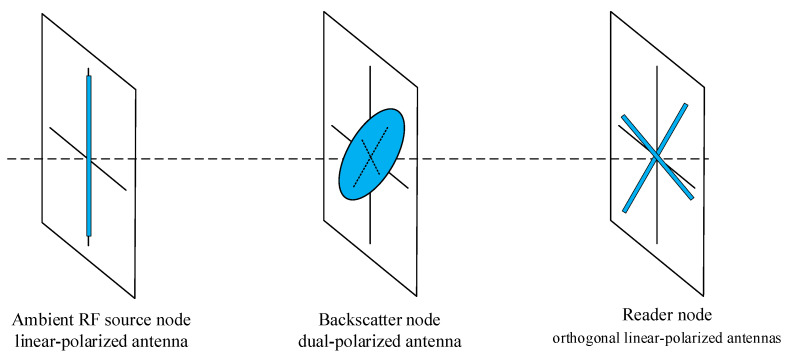
Relative antenna positions of each node.

**Figure 7:**
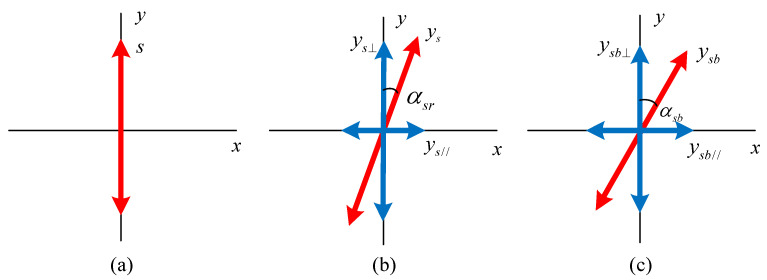
Signal polarizations at different nodes. (**a**) ambient RF source node transmit signal; (**b**) reader node received signal from ambient RF source node; (**c**) backscatter node received signal from ambient RF source node.

In the elementary bistatic AmBC system model as shown in Figure 1, there are three signal paths: from the ambient RF source node to the reader node, from the ambient RF source node to the backscatter node, and from the backscatter node to the reader node. Because polarization patterns are utilized, the signal frequently undergoes uncorrelated fading, so two independent channel coefficients are considered for each path. Let $h_{sr⊥}$ and $h_{sr//}$ denote the channel coefficients of the path between the ambient RF source node and the reader node, $h_{sb⊥}$ and $h_{sb//}$ denote the channel coefficients of the path between the ambient RF source node and the backscatter node, and $h_{br⊥}$ and $h_{br//}$ denote the channel coefficients of the path between the backscatter node and the reader node. Note that the notation refers to the orthogonality of the antennas, but they may not consistently exhibit perfect vertical or horizontal polarization. Therefore, one of the two orthogonal patterns is designated as a “reference” vertical polarization pattern, and the other is designated as a “reference” horizontal polarization pattern at the backscatter node and the reader node. Moreover, in most practical situations, it is difficult to ensure that the transmit antenna of the ambient RF source node and the receive antenna of the reader node are aligned, despite both being vertical linear-polarized. There are also many reflective objects in real environments, which lead to polarization mismatch the received signal at the reader node. The polarization mismatch depends on the angle between the transmit antenna and the receive antennas. As per the system model shown in Figure 1, we assume that the ambient RF source transmit antenna is accurately vertical linear-polarized. The two orthogonal linear-polarized receive antennas at the reader node can be in any rotation direction but must lie in the same plane, perpendicular to the direction pf electromagnetic wave propagation from the ambient RF source transmit antenna, as shown in Figure 6. Figure 7a shows the polarization of the transmitted signal at the ambient RF source node, and Figure 7b defines *α_sr_* as the included angle between the received signal at the reader node and its “reference” vertical polarized antenna, accounting for the effect of the antenna angle and reflection off of environment objects. Then, the received signal from the ambient RF source node at the two orthogonal linear-polarized receive antennas of the reader node can be represented as

(1) $$\left\{\begin{array}{l} y_{s⊥}(t)=h_{sr⊥}s(t)\cdot \cos\alpha_{sr}\\ y_{s//}(t)=h_{sr//}s(t)\cdot \sin\alpha_{sr} \end{array}\right.,$$

where *s*(*t*) is the ambient RF signal with average power *P_s_*, which can be assumed to be a complex Gaussian signal.

Similarly, let *α_sb_* represent the included angle between the received signal at the backscatter node and the “reference” vertical polarization pattern of the backscatter node, as shown in Figure 7c. Then, the received signal at the two orthogonal polarization patterns of the dual-polarized antenna of the backscatter node from the ambient RF source node can be represented as

(2) $$\left\{\begin{array}{l} y_{sb⊥}(t)=h_{sb⊥}s(t)\cdot \cos\alpha_{sb}\\ y_{sb//}(t)=h_{sb//}s(t)\cdot \sin\alpha_{sb} \end{array}\right..$$

As the backscatter node and the reader node are generally close to each other compared to the distance from the ambient RF source node to them, we assume that the polarization patterns of the backscatter dual-polarized antenna and the reader orthogonal polarization antennas are well aligned. Then, the received signal at the two orthogonal linear-polarized receive antennas of the reader node from the backscatter node can be represented as

(3) $$\left\{\begin{array}{l} y_{b⊥}(t)=\eta h_{sb⊥}h_{br⊥}b_{⊥}s(t)\cdot \cos\alpha_{sb}\\ y_{b//}(t)=\eta h_{sb//}h_{br//}b_{//}s(t)\cdot \sin\alpha_{sb} \end{array}\right.,$$

where *η* denotes the reflection coefficient of the backscatter node and $b_{⊥}$ and $b_{//}$ denote the original information generated by the microcontroller on different polarization patterns, which will be described in detail in subsequent sections.

Thus, the entire received signal at the two orthogonal linear-polarized receive antennas of the reader node can be represented as

(4) $$\left\{\begin{array}{l} y_{⊥}(t)=h_{sr⊥}s(t)\cdot \cos\alpha_{sr}+\eta h_{sb⊥}h_{br⊥}b_{⊥}s(t)\cdot \cos\alpha_{sb}+w_{⊥}(t)\\ y_{//}(t)=h_{sr//}s(t)\cdot \sin\alpha_{sr}+\eta h_{sb//}h_{br//}b_{//}s(t)\cdot \sin\alpha_{sb}+w_{//}(t) \end{array}\right.,$$

where $w_{⊥}(t)$ and $w_{//}(t)$ are independent zero-mean additive white Gaussian noise (AWGN) components for each antenna, each with a variance *N_w_*, i.e., $w_{⊥}(t),w_{//}(t)∼CN(0,N_{w})$.

In reality, *α_sr_* and *α_sb_* are random, with a maximum value range of [−90°, 90°], and they are hard to measure in complex real-world environments. They can be modeled and approximated as random variables. It is reasonable to include these angles as a part of the channel state information (CSI), as small-scale fading mainly represents multi-path effects and included angles are caused mainly by polarization mismatch [36].

The DDB node structure is simple and requires only one dual-polarized antenna. However, it also has a significant disadvantage in that when *α_sb_* is close to 0° or ±90°, one of the polarization patterns of the dual-polarized antenna will receive very low power, adversely affecting backscatter signal and information bit detection. Furthermore, when the ambient RF source node is significantly distant from both the backscatter node and the reader node, it may not be possible to establish a backscatter path in each direction. Thus, we consider another structure for the backscatter node in the DPAm system.

### 2.3. Polarization Conversion Backscatter (PCB)

Considering a large-scale factory or agricultural environment, the distance between the backscatter node (e.g., a smart tag) and the reader node (e.g., a hand-held central controller) is on the scale of several meters, while the distance between the ambient RF source node (e.g., a base station) and the backscatter node or the reader node is 100 m or more. Therefore, the direction from which the ambient RF signal arrives is difficult to determined. If the ground plane of the backscatter antenna is oriented towards the arrival direction of the ambient RF signal, it may fail to establish a backscatter path with the reader node. To address this issue, we proposed a PCB backscatter node structure tailored for these situatuins.

**Figure 8:**
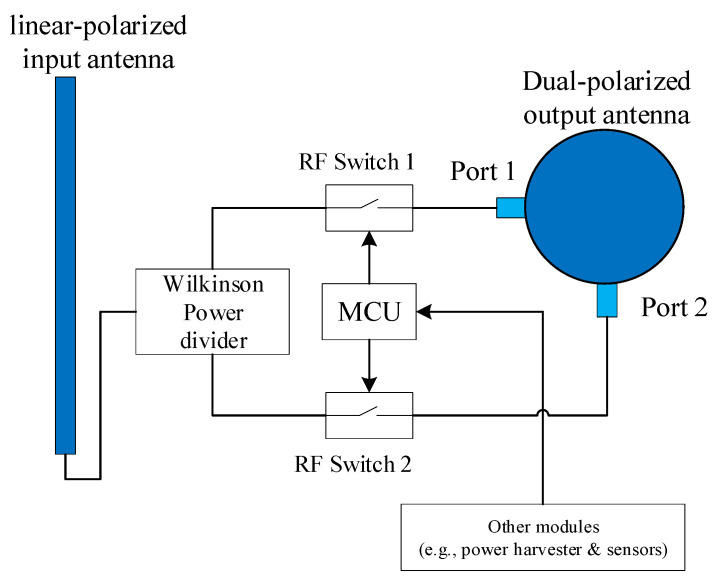
Backscatter node with PCB structure.

In this case, inspired by the polarization conversion method mentioned in [23], we design another dual-polarization backscatter node structure based on polarization conversion. This node has a linear-polarized input antenna and a dual-polarized output antenna, as shown in Figure 8. (In [23], the output antenna is circularly polarized; it is also suitable for this backscatter node, while the reader node should be equipped with antenna that have a corresponding polarization pattern). The input antenna should be omnidirectional to ensure that it receives sufficient ambient RF signal power for backscatter communication. Similar to the DDB node structure, each port of the output antenna connects separately to an RF switch, and the RF switches are controlled by the microcontroller. In this structure, modulation of information bits is achieved by converting the received signal power from the linear-polarized input antenna to the corresponding polarization pattern of the dual-polarized output antenna, rather than by choosing matched or mismatched impedance. When one of the RF switches is on, the received signal power is transformed to the corresponding polarization pattern of the dual-polarized output antenna and backscattering a signal that represents bit ‘1’. Conversely, when the RF switch is off, none of the received power is converted, representing bit ‘0’. However, when if both polarization patterns need to backscatter bit ‘1’ backscatter bit ‘1’ simultaneously, the received signal power should be equally divided between them. Therefore, a passive Wilkinson power divider is essential for this structure. The control signal is also provided by a low-power microcontroller, which modulates information bits on the backscatter signal.

In the case of the PCB node structure, the backscatter node first receives the ambient RF signal at the linear-polarized input antenna. As the input antenna is single-polarized, here *α_sb_* denotes the included angle between the direction of the received signal at the backscatter node and the linear-polarized input antenna. Then, the received signal at the input antenna of the backscatter node from the ambient RF source node can be represented as

(5) $$y_{bi}(t)=h_{sb⊥}s(t)\cdot \cos\alpha_{sb}.$$

As the Wilkinson power divider is used to divide power into two equal parts, the received signal at the two orthogonal linear-polarized receive antennas of the reader node from the backscatter node can be represented as

(6) $$\left\{\begin{array}{l} y_{b⊥}(t)=\frac{1}{2}\eta h_{sb⊥}h_{br⊥}b_{⊥}s(t)\cdot \cos\alpha_{sb}\\ y_{b//}(t)=\frac{1}{2}\eta h_{sb⊥}h_{br//}b_{//}s(t)\cdot \cos\alpha_{sb} \end{array}\right..$$

Thus, the entire received signal at the two orthogonal linear-polarized receive antennas of the reader node can be represented as

(7) $$\left\{\begin{array}{l} y_{⊥}(t)=h_{sr⊥}s(t)\cdot \cos\alpha_{sr}+\frac{1}{2}\eta h_{sb⊥}h_{br⊥}b_{⊥}s(t)\cdot \cos\alpha_{sb}+w_{⊥}(t)\\ y_{//}(t)=h_{sr//}s(t)\cdot \sin\alpha_{sr}+\frac{1}{2}\eta h_{sb⊥}h_{br//}b_{//}s(t)\cdot \cos\alpha_{sb}+w_{//}(t) \end{array}\right..$$

The PCB node structure is more complex than the DDB node structure and requires two antennas. Moreover, to avoid the inter-antenna coupling effect, the ground plane of the dual-polarized output antenna should be oriented towards the input antenna, which imposes stricter design requirements. However, in contrast to the DDB node structure, it is possible to keep the backscatter signal power equal at different polarization patterns and to reduce the influence of the arrival direction of the ambient RF signal. When *α_sb_* is close to ±90°, the received power at the input antenna will still be very low; however, this scenario represents an extreme case. Therefore, the PCB node structure offers greater fairness in signal distribution than the DDB node structure.

## 3. Parallel Backscatter Mode with Differential Coding

Based on the DPAm system discussed above, the simplest backscatter scheme is to utilize different polarization patterns to transmit different information bits independently. As the two polarization patterns are uncorrelated, the DPAm system provides an additional path for transmitting the information bits. Hence, we propose implementing differential coding across different polarization patterns.

### 3.1. Differential Coding Scheme

**Figure 9:**
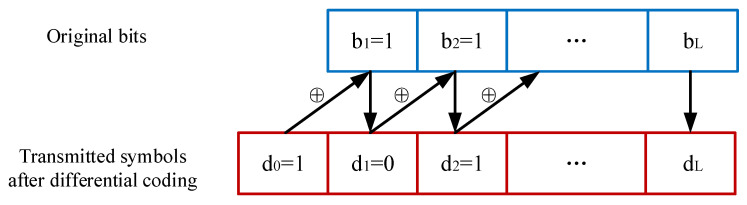
Frame form of the differential coding scheme.

We assume a frequency-flat and block-fading channel model as in [24,25,26,27]. This model implies that each channel coefficient remains constant within a single coherent frame but changes independently in the adjacent frame. To confirm the relative relationships among the backscatter information bits, the first symbol of the differential coding sequence is fixed as *d*_0_ = 1 and the *l*-th subsequent symbol of the differential coding sequence is denoted by $d_{l}=d_{l-1}⊕b_{l},l\in \{1,\cdots ,L\}$ as shown in Figure 9, where ⊕ represents addition modulo 2. Thus, one frame consists of *L* + 1 time slots dedicated to backscatter symbols.

To further process the received signal on digital hardware such as a microcontroller, each $d_{l⊥}$ or $d_{l//}$ time slot should be sampled at the Nyquist rate with a sample count of *N*. This strategy ensures that each backscatter symbol time slot will remain consistent for *N* samples of the ambient RF signal. Thus, we denote the received vectors in the *l*-th backscatter symbol time slot at different polarization patterns of the reader node as $y_{l⊥}=\{y_{l⊥}[1],\cdots ,y_{l⊥}[n],\cdots ,y_{l⊥}[N]\}$ and $y_{l//}=\{y_{l//}[1],\cdots ,y_{l//}[n],\cdots ,y_{l//}[N]\}$.

In the DDB node structure, the received signal (4) can be formulated as

(8) $$\left\{\begin{array}{l} y_{l⊥}[n]=h_{0⊥}^{DDB}s[n]+w_{⊥}[n],\;\;\;\;d_{l⊥}=0\\ y_{l⊥}[n]=h_{1⊥}^{DDB}s[n]+w_{⊥}[n],\;\;\;\;d_{l⊥}=1\\ y_{l//}[n]=h_{0//}^{DDB}s[n]+w_{//}[n],\;\;\;\;d_{l//}=0\\ y_{l//}[n]=h_{1//}^{DDB}s[n]+w_{//}[n],\;\;\;\;d_{l//}=1 \end{array}\right.,$$

where $h_{0⊥}^{DDB}≜h_{sr⊥}\cos\alpha_{sr}$, $h_{1⊥}^{DDB}≜h_{sr⊥}\cos\alpha_{sr}+\eta h_{sb⊥}h_{br⊥}\cos\alpha_{sb}$, $h_{0//}^{DDB}≜h_{sr//}\sin\alpha_{sr}$, $h_{1//}^{DDB}≜h_{sr//}\sin\alpha_{sr}+\eta h_{sb//}h_{br//}\sin\alpha_{sb}$.

If we assume that the ambient RF signal *s*[*n*] is a zero-mean complex Gaussian signal, i.e., $s[n]∼CN(0,P_{s})$, according to (8) it has

(9) $$\left\{\begin{array}{l} y_{l⊥}[n]∼CN(0,\sigma_{0⊥}^{2}{}^{DDB}),\;\;\;\;d_{l⊥}=0\\ y_{l⊥}[n]∼CN(0,\sigma_{1⊥}^{2}{}^{DDB}),\;\;\;\;d_{l⊥}=1\\ y_{l//}[n]∼CN(0,\sigma_{0//}^{2}{}^{DDB}),\;\;\;\;d_{l//}=0\\ y_{l//}[n]∼CN(0,\sigma_{1//}^{2}{}^{DDB}),\;\;\;\;d_{l//}=1 \end{array}\right.,$$

where $\sigma_{0⊥}^{2}{}^{DDB}≜$${\left|h_{0⊥}^{DDB}\right|}^{2}P_{s}+N_{w}$, $\sigma_{1⊥}^{2}{}^{DDB}≜$${\left|h_{1⊥}^{DDB}\right|}^{2}P_{s}+N_{w}$, $\sigma_{0//}^{2}{}^{DDB}≜$${\left|h_{0//}^{DDB}\right|}^{2}P_{s}+N_{w}$, $\sigma_{1//}^{2}{}^{DDB}≜$${\left|h_{1//}^{DDB}\right|}^{2}P_{s}+N_{w}$.

In the PCB node structure, similarly, we have

(10) $$\left\{\begin{array}{l} y_{l⊥}[n]=h_{0⊥}^{PCB}s[n]+w_{⊥}[n],\;\;\;\;d_{l⊥}=0\\ y_{l⊥}[n]=h_{1⊥}^{PCB}s[n]+w_{⊥}[n],\;\;\;\;d_{l⊥}=1\\ y_{l//}[n]=h_{0//}^{PCB}s[n]+w_{//}[n],\;\;\;\;d_{l//}=0\\ y_{l//}[n]=h_{1//}^{PCB}s[n]+w_{//}[n],\;\;\;\;d_{l//}=1 \end{array}\right.,$$

where $h_{0⊥}^{PCB}≜h_{sr⊥}\cos\alpha_{sr}$, $h_{1⊥}^{PCB}≜h_{sr⊥}\cos\alpha_{sr}+\frac{1}{2}\eta h_{sb⊥}h_{br⊥}\cos\alpha_{sb}$, $h_{0//}^{PCB}≜h_{sr//}\sin\alpha_{sr}$, $h_{1//}^{PCB}≜h_{sr//}\sin\alpha_{sr}+\frac{1}{2}\eta h_{sb⊥}h_{br//}\cos\alpha_{sb}$.

Also, we have

(11) $$\left\{\begin{array}{l} y_{l⊥}[n]∼CN(0,\sigma_{0⊥}^{2}{}^{PCB}),\;\;\;\;d_{l⊥}=0\\ y_{l⊥}[n]∼CN(0,\sigma_{1⊥}^{2}{}^{PCB}),\;\;\;\;d_{l⊥}=1\\ y_{l//}[n]∼CN(0,\sigma_{0//}^{2}{}^{PCB}),\;\;\;\;d_{l//}=0\\ y_{l//}[n]∼CN(0,\sigma_{1//}^{2}{}^{PCB}),\;\;\;\;d_{l//}=1 \end{array}\right.,$$

where $\sigma_{0⊥}^{2}{}^{PCB}≜$${\left|h_{0⊥}^{PCB}\right|}^{2}P_{s}+N_{w}$, $\sigma_{1⊥}^{2}{}^{PCB}≜$${\left|h_{1⊥}^{PCB}\right|}^{2}P_{s}+N_{w}$, $\sigma_{0//}^{2}{}^{PCB}≜$${\left|h_{0//}^{PCB}\right|}^{2}P_{s}+N_{w}$, $\sigma_{1//}^{2}{}^{PCB}≜$${\left|h_{1//}^{PCB}\right|}^{2}P_{s}+N_{w}$.

### 3.2. Joint-Energy Detector

From (8) and (9), it can be seen that the received signal power is significant when $d_{l⊥}$ or $d_{l//}$ represent different bits. Thus, let $Z_{l⊥}={\left\|y_{l⊥}\right\|}^{2}$ and $Z_{l//}={\left\|y_{l//}\right\|}^{2}$ denote the power of the backscatter symbol in each frame.

Next, we propose the use of a joint-energy detector for the parallel backscatter mode in the DPAm system. According to the differential coding scheme, each original information bit is dependent on the adjacent backscatter symbols. Let $T_{⊥}$ and $T_{//}$ denote the detection thresholds for different polarization patterns. Given that all the channel coefficients remain constant within a frame, when the original information bit $b_{⊥}$ or $b_{//}$ is bit ‘0’, the current backscatter symbol and last backscatter symbol should be the same. Consequently, the power of the current backscatter symbol and that of the last backscatter symbol should have the same relationship to their detection thresholds. Similarly, when the original information bit $b_{⊥}$ or $b_{//}$ is bit ‘1’, the current backscatter symbol and last backscatter symbol should be the inverse if each other. Consequently, the power of the current backscatter symbol and that of the last backscatter symbol should have inverse relationships to their detection thresholds. Therefore, we have

(1) if $Z_{(l-1)⊥}\leq T_{⊥}$ and $Z_{l⊥}\leq T_{⊥}$, or $Z_{(l-1)⊥}>T_{⊥}$ and $Z_{l⊥}>T_{⊥}$, the *l*-th original information bit is determined to be ${\hat{b}}_{l⊥}=0$;
(2) if $Z_{(l-1)⊥}>T_{⊥}$ and $Z_{l⊥}\leq T_{⊥}$, or $Z_{(l-1)⊥}\leq T_{⊥}$ and $Z_{l⊥}>T_{⊥}$, the *l*-th original information bit is determined to be ${\hat{b}}_{l⊥}=1$;
(3) if $Z_{(l-1)//}\leq T_{//}$ and $Z_{l//}\leq T_{//}$, or $Z_{(l-1)//}>T_{//}$ and $Z_{l//}>T_{//}$, the *l*-th original information bit is determined to be ${\hat{b}}_{l//}=0$;
(4) if $Z_{(l-1)//}>T_{//}$ and $Z_{l//}\leq T_{//}$, or $Z_{(l-1)//}\leq T_{//}$ and $Z_{l//}>T_{//}$, the *l*-th original information bit is determined to be ${\hat{b}}_{l//}=1$.

To confirm the optimal $T_{⊥}$ and $T_{//}$, we further analyze $Z_{l⊥}$ and $Z_{l//}$. Combining the definition of $Z_{l⊥}$, $Z_{l//}$ and $y_{l⊥}$, $y_{l//}$, it can be easily seen that

(12) $$\left\{\begin{array}{l} Z_{l⊥}=\sum_{n=1}^{N}{\left|y_{l⊥}[n]\right|}^{2}\\ Z_{l//}=\sum_{n=1}^{N}{\left|y_{l//}[n]\right|}^{2} \end{array}\right..$$

According to (9), ${\left|y_{l⊥}[n]\right|}^{2}$ and ${\left|y_{l//}[n]\right|}^{2}$ are central chi-square random variables with 2 degrees of freedom. Therefore, they have the means $\sigma_{i⊥}^{2}{}^{DDB}$, $\sigma_{j//}^{2}{}^{DDB}$ and the variances $\sigma_{i⊥}^{4}{}^{DDB}$, $\sigma_{j//}^{4}{}^{DDB}$ for the DDB node structure, and the means $\sigma_{i⊥}^{2}{}^{PCB}$, $\sigma_{j//}^{2}{}^{PCB}$ and the variances $\sigma_{i⊥}^{4}{}^{PCB}$, $\sigma_{j//}^{4}{}^{PCB}$ for the PCB node structure when $d_{l⊥}=i$ and $d_{l//}=j$, separately.

In the DDB node structure, by the central limit theorem, the distributions of $Z_{l⊥}$ and $Z_{l//}$ separately asymptotically approach a Gaussian distribution. We denote $Z_{l⊥|i}$ and $Z_{l//|j}$ as the power of the backscatter symbol when $d_{l⊥}=i$ and $d_{l//}=j$. We then have

(13) $$\left\{\begin{array}{l} Z_{l⊥|i}∼N(N\sigma_{i⊥}^{2}{}^{DDB},N\sigma_{i⊥}^{4}{}^{DDB}),\;\;\;\;\;i=0,1\\ Z_{l//|j}∼N(N\sigma_{j//}^{2}{}^{DDB},N\sigma_{j//}^{4}{}^{DDB}),\;\;\;\;j=0,1 \end{array}\right..$$

Next, we can obtain the optimal detection threshold by letting $f_{Z_{l⊥|0}}(T_{⊥}^{DDB})=f_{Z_{l⊥|1}}(T_{⊥}^{DDB})$ and $f_{Z_{l//|0}}(T_{//}^{DDB})=f_{Z_{l//|1}}(T_{//}^{DDB})$, which means the optimal detection threshold let the probability density of the power of the backscatter symbol when the backscatter symbol $d_{l⊥}$ or $d_{l//}$ represents bit ‘1’ is equal to the probability density of the power of the backscatter symbol when the backscatter symbol $d_{l⊥}$ or $d_{l//}$ represents bit ‘0’. Thus, the optimal detection threshold is designed to divide the range of the backscatter symbol power to minimize the probability of error. After some computation, we obtain

(14) $$\left\{\begin{array}{l} T_{⊥}^{DDB}=\frac{N\sigma_{0⊥}^{2}{}^{DDB}\sigma_{1⊥}^{2}{}^{DDB}}{\sigma_{0⊥}^{2}{}^{DDB}+\sigma_{1⊥}^{2}{}^{DDB}}[1+\sqrt{1+\frac{2(\sigma_{1⊥}^{2}{}^{DDB}+\sigma_{0⊥}^{2}{}^{DDB})}{N(\sigma_{1⊥}^{2}{}^{DDB}-\sigma_{0⊥}^{2}{}^{DDB})}\ln\frac{\sigma_{1⊥}^{2}{}^{DDB}}{\sigma_{0⊥}^{2}{}^{DDB}}}]\\ T_{//}^{DDB}=\frac{N\sigma_{0//}^{2}{}^{DDB}\sigma_{1//}^{2}{}^{DDB}}{\sigma_{0//}^{2}{}^{DDB}+\sigma_{1//}^{2}{}^{DDB}}[1+\sqrt{1+\frac{2(\sigma_{1//}^{2}{}^{DDB}+\sigma_{0//}^{2}{}^{DDB})}{N(\sigma_{1//}^{2}{}^{DDB}-\sigma_{0//}^{2}{}^{DDB})}\ln\frac{\sigma_{1//}^{2}{}^{DDB}}{\sigma_{0//}^{2}{}^{DDB}}}] \end{array}\right..$$

When *N* is a large value (commonly larger than 10), (11) could be approximated as

(15) $$\left\{\begin{array}{l} T_{⊥}^{DDB}=\frac{N\sigma_{0⊥}^{2}{}^{DDB}\sigma_{1⊥}^{2}{}^{DDB}}{\sigma_{0⊥}^{2}{}^{DDB}+\sigma_{1⊥}^{2}{}^{DDB}}\\ T_{//}^{DDB}=\frac{N\sigma_{0//}^{2}{}^{DDB}\sigma_{1//}^{2}{}^{DDB}}{\sigma_{0//}^{2}{}^{DDB}+\sigma_{1//}^{2}{}^{DDB}} \end{array}\right..$$

After the optimal detection threshold has been obtained, it is feasible to obtain the analytical bit error rate (BER). Considering the decision regions mentioned above, we also assume that the probabilities of the backscatter symbol representing bit ‘0’ or bit ‘1’ are equal. Then, the error probability on each polarization pattern can be represented as

(16) $$\begin{array}{l} \left\{\begin{array}{ll} P_{⊥}^{DDB} & =\frac{1}{2}\{\frac{1}{2}[P(Z_{(l-1)⊥|0}\leq T_{⊥}^{DDB},Z_{l⊥|1}\leq T_{⊥}^{DDB})+P(Z_{(l-1)⊥|1}\leq T_{⊥}^{DDB},Z_{l⊥|0}\leq T_{⊥}^{DDB})]\\ & +\frac{1}{2}[P(Z_{(l-1)⊥|0}>T_{⊥}^{DDB},Z_{l⊥|1}>T_{⊥}^{DDB})+P(Z_{(l-1)⊥|1}>T_{⊥}^{DDB},Z_{l⊥|0}>T_{⊥}^{DDB})]\\ & +\frac{1}{2}[P(Z_{(l-1)⊥|0}>T_{⊥}^{DDB},Z_{l⊥|0}\leq T_{⊥}^{DDB})+P(Z_{(l-1)⊥|0}\leq T_{⊥}^{DDB},Z_{l⊥|0}>T_{⊥}^{DDB})]\\ & +\frac{1}{2}[P(Z_{(l-1)⊥|1}>T_{⊥}^{DDB},Z_{l⊥|1}\leq T_{⊥}^{DDB})+P(Z_{(l-1)⊥|1}\leq T_{⊥}^{DDB},Z_{l⊥|1}>T_{⊥}^{DDB})]\}\\ P_{//}^{DDB} & =\frac{1}{2}\{\frac{1}{2}[P(Z_{(l-1)//|0}\leq T_{//}^{DDB},Z_{l//|1}\leq T_{//}^{DDB})+P(Z_{(l-1)//|1}\leq T_{//}^{DDB},Z_{l//|0}\leq T_{//}^{DDB})]\\ & +\frac{1}{2}[P(Z_{(l-1)//|0}>T_{//}^{DDB},Z_{l//|1}>T_{//}^{DDB})+P(Z_{(l-1)//|1}>T_{//}^{DDB},Z_{l//|0}>T_{//}^{DDB})]\\ & +\frac{1}{2}[P(Z_{(l-1)//|0}>T_{//}^{DDB},Z_{l//|0}\leq T_{//}^{DDB})+P(Z_{(l-1)//|0}\leq T_{//}^{DDB},Z_{l//|0}>T_{//}^{DDB})]\\ & +\frac{1}{2}[P(Z_{(l-1)//|1}>T_{//}^{DDB},Z_{l//|1}\leq T_{//}^{DDB})+P(Z_{(l-1)//|1}\leq T_{//}^{DDB},Z_{l//|1}>T_{//}^{DDB})]\} \end{array}\right. \end{array},$$

while $Z_{(l-1)⊥}$ and $Z_{l⊥}$, $Z_{(l-1)//}$ and $Z_{l//}$ are independent, from (13) we have

(17) $$\left\{\begin{array}{l} P(Z_{l⊥|i}\geq T_{⊥}^{DDB})=Q(\frac{T_{⊥}^{DDB}-N\sigma_{i⊥}^{2}{}^{DDB}}{\sqrt{N}\sigma_{i⊥}^{2}{}^{DDB}}),\;\;\;\;\;i=0,1\\ P(Z_{l//|j}\geq T_{//}^{DDB})=Q(\frac{T_{//}^{DDB}-N\sigma_{j//}^{2}{}^{DDB}}{\sqrt{N}\sigma_{j//}^{2}{}^{DDB}}),\;\;\;\;j=0,1 \end{array}\right.,$$

where *Q*(*x*) is the *Q*-function, which is denoted by $Q(x)=\frac{1}{\sqrt{2\pi }}\int_{x}^{\infty }e^{-\frac{t^{2}}{2}}dt$.

Combining (16) and (17), after some computation we obtain

(18) $$\left\{\begin{array}{l} P_{⊥}^{DDB}=\frac{1}{2}-\frac{1}{2}{\left[Q(\frac{T_{⊥}^{DDB}-N\sigma_{0⊥}^{2}{}^{DDB}}{\sqrt{N}\sigma_{0⊥}^{2}{}^{DDB}})-Q(\frac{T_{⊥}^{DDB}-N\sigma_{1⊥}^{2}{}^{DDB}}{\sqrt{N}\sigma_{1⊥}^{2}{}^{DDB}})\right]}^{2}\\ P_{//}^{DDB}=\frac{1}{2}-\frac{1}{2}{\left[Q(\frac{T_{//}^{DDB}-N\sigma_{0//}^{2}{}^{DDB}}{\sqrt{N}\sigma_{0//}^{2}{}^{DDB}})-Q(\frac{T_{//}^{DDB}-N\sigma_{1//}^{2}{}^{DDB}}{\sqrt{N}\sigma_{1//}^{2}{}^{DDB}})\right]}^{2} \end{array}\right.,$$

Taking (15) into (18), we obtain the analytical BER as follows:

(19) $$\left\{\begin{array}{l} P_{⊥}^{DDB}=2Q(\frac{\sqrt{N}\left|\sigma_{0⊥}^{2}{}^{DDB}-\sigma_{1⊥}^{2}{}^{DDB}\right|}{\sigma_{0⊥}^{2}{}^{DDB}+\sigma_{1⊥}^{2}{}^{DDB}})[1-Q(\frac{\sqrt{N}\left|\sigma_{0⊥}^{2}{}^{DDB}-\sigma_{1⊥}^{2}{}^{DDB}\right|}{\sigma_{0⊥}^{2}{}^{DDB}+\sigma_{1⊥}^{2}{}^{DDB}})]\\ P_{//}^{DDB}=2Q(\frac{\sqrt{N}\left|\sigma_{0//}^{2}{}^{DDB}-\sigma_{1//}^{2}{}^{DDB}\right|}{\sigma_{0//}^{2}{}^{DDB}+\sigma_{1//}^{2}{}^{DDB}})[1-Q(\frac{\sqrt{N}\left|\sigma_{0//}^{2}{}^{DDB}-\sigma_{1//}^{2}{}^{DDB}\right|}{\sigma_{0//}^{2}{}^{DDB}+\sigma_{1//}^{2}{}^{DDB}})] \end{array}\right..$$

If the backscatter node transmits backscatter symbols with data rate *R_b_* on each polarization pattern, i.e., each RF switch shown in Figure 5 switches *R_b_* times per second, the channel capacity for backscatter symbols is

(20) $$\left\{\begin{array}{l} C_{⊥}^{DDB}=R_{b}[1+P_{⊥}^{DDB}\log_{2}P_{⊥}^{DDB}+(1-P_{⊥}^{DDB})\log_{2}(1-P_{⊥}^{DDB})]\\ C_{//}^{DDB}=R_{b}[1+P_{//}^{DDB}\log_{2}P_{//}^{DDB}+(1-P_{//}^{DDB})\log_{2}(1-P_{//}^{DDB})] \end{array}\right..$$

Therefore, the sum throughput of the DDB structure node is

(21) $$R^{DDB}=C_{⊥}^{DDB}+C_{//}^{DDB}.$$

In the PCB node structure, by similar analysis, we can also obtain the approximate optimal detection threshold as

(22) $$\left\{\begin{array}{l} T_{⊥}^{PCB}=\frac{N\sigma_{0⊥}^{2}{}^{PCB}\sigma_{1⊥}^{2}{}^{PCB}}{\sigma_{0⊥}^{2}{}^{PCB}+\sigma_{1⊥}^{2}{}^{PCB}}\\ T_{//}^{PCB}=\frac{N\sigma_{0//}^{2}{}^{PCB}\sigma_{1//}^{2}{}^{PCB}}{\sigma_{0//}^{2}{}^{PCB}+\sigma_{1//}^{2}{}^{PCB}} \end{array}\right.,$$

and the analytical BER as

(23) $$\left\{\begin{array}{l} P_{⊥}^{PCB}=2Q(\frac{\sqrt{N}\left|\sigma_{0⊥}^{2}{}^{PCB}-\sigma_{1⊥}^{2}{}^{PCB}\right|}{\sigma_{0⊥}^{2}{}^{PCB}+\sigma_{1⊥}^{2}{}^{PCB}})[1-Q(\frac{\sqrt{N}\left|\sigma_{0⊥}^{2}{}^{PCB}-\sigma_{1⊥}^{2}{}^{PCB}\right|}{\sigma_{0⊥}^{2}{}^{PCB}+\sigma_{1⊥}^{2}{}^{PCB}})]\\ P_{//}^{PCB}=2Q(\frac{\sqrt{N}\left|\sigma_{0//}^{2}{}^{PCB}-\sigma_{1//}^{2}{}^{PCB}\right|}{\sigma_{0//}^{2}{}^{PCB}+\sigma_{1//}^{2}{}^{PCB}})[1-Q(\frac{\sqrt{N}\left|\sigma_{0//}^{2}{}^{PCB}-\sigma_{1//}^{2}{}^{PCB}\right|}{\sigma_{0//}^{2}{}^{PCB}+\sigma_{1//}^{2}{}^{PCB}})] \end{array}\right..$$

Therefore, we also have the channel capacity for backscatter symbols as

(24) $$\left\{\begin{array}{l} C_{⊥}^{PCB}=R_{b}[1+P_{⊥}^{PCB}\log_{2}P_{⊥}^{PCB}+(1-P_{⊥}^{PCB})\log_{2}(1-P_{⊥}^{PCB})]\\ C_{//}^{PCB}=R_{b}[1+P_{//}^{PCB}\log_{2}P_{//}^{PCB}+(1-P_{//}^{PCB})\log_{2}(1-P_{//}^{PCB})] \end{array}\right..$$

The sum throughput of the PCB structure node is

(25) $$R^{PCB}=C_{⊥}^{PCB}+C_{//}^{PCB}.$$

## 4. Simultaneous Backscatter Mode with Manchester Coding

Although the parallel backscatter mode and the joint-energy detector are simple, their operation requires various signal parameters. As it is difficult to precisely determine signal variance, we further consider detectors that do not require estimation of CSI. Hence, we propose adopting Manchester coding on both polarization patterns. We also propose switching from parallel backscatter mode to simultaneous backscatter mode. Finally, we propose using a simple power average detector and an unsupervised machine learning-based detector that uses K-means clustering.

### 4.1. Manchester Coding Scheme

As channel coefficients generally have similar variances in similar environments, the variance of the ambient RF signal will be larger than the variance of the channel coefficients, especially in the case of high SNR. Thus, the effect of the ambient RF signal will be greater. In contrast to the significant power fluctuations in the ambient RF signal between adjacent symbol time slots, the ambient RF signal *s*(*t*) is consistent for different polarization patterns in the same time slot. It is thus better to encode information in the same backscatter symbol time slot in order to improve the stability of the DPAm system. In order to further exploit the advantages of DPAm and simplify the detection schemes, we propose the use of a simultaneous backscatter mode coupled with a Manchester coding scheme for the DPAm system.

**Figure 10:**
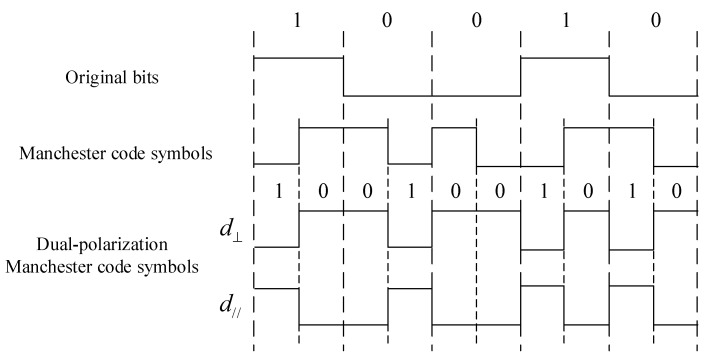
Encoding samples of the Manchester coding scheme.

Manchester code is a simple block code that separately maps bit ‘0’ and ‘1’ into ‘01’ and ‘10’. It has been widely used in RFID [37,38,39] and AmBC systems [26,40,41]. In this paper, we adopt the IEEE 802.3 standard [42] convention for Manchester coding, wherein the original bit ‘0’ is represented by ‘10’ and the original bit ‘1’ is represented by ‘01’. Then, we modulate the first half of the Manchester code symbol on one of the polarization patterns, i.e., $d_{⊥}$, and the second half on the other polarization pattern, i.e., $d_{//}$, as shown in Figure 10. In other words, the adoption of Manchester coding means that only one of the polarization patterns will be activated to backscatter at all times.

In contrast to a SPAm system with Manchester coding, the proposed DPAm system uses two polarization patterns to modulate two bits of one Manchester symbol and thus can theoretically double the data rate. We also consider relative data rate gain in the simulation results.

**Figure 11:**
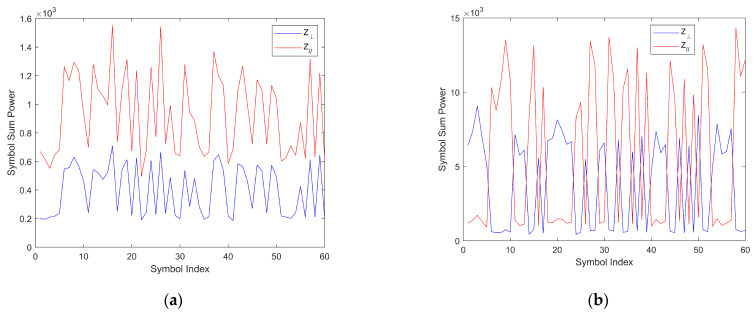
Samples of the backscatter symbol power on the two polarization patterns in one frame. (**a**) with the same trends, (**b**) with opposite trends.

As small-scale multipath fading can both increase and decrease the entire received signal, and as the effect of the unknown included angle leads to unequal and random direct path received power on the two polarization patterns ${\left|y_{s⊥}\right|}^{2}$ and ${\left|y_{s//}\right|}^{2}$, there is a challenge in this system. On the same polarization pattern, the received signal power for bit ‘1’ may not always be higher than that for bit ‘0’. Moreover, on different polarization patterns, the average received signal power will be uncertain, leading to a “power imbalance problem” in the DPAm system. In parallel backscatter mode, the two polarization patterns are treated as independent, which avoids the power imbalance problem; however, the power imbalance problem presents challenges in simultaneous backscatter mode. To clearly demonstrate the power imbalance problem, two samples of the backscatter symbol power in one frame are shown in Figure 11. It can be readily seen that the backscatter symbol power on different polarization patterns has different means and variances. In addition, the power trends for each backscatter symbol in different polarization patterns can be either the same and or opposite each other when using Manchester coding. Thus, it is not possible to directly compare the received power on different polarization patterns or to compute a detection threshold as in Section 3.2.

**Figure 12:**
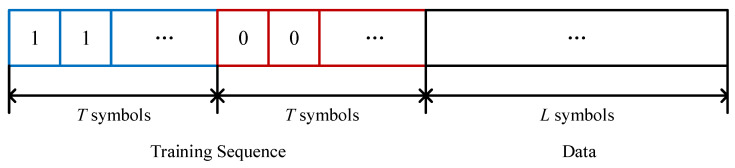
Frame form of the Manchester coding scheme.

As CSI estimation is difficult in such a low-power system and in complex environments, indirect estimation of power relationships using known symbols is essential. For instance, we combine *T* Manchester coding symbols representing ‘1’, *T* Manchester coding symbols representing ‘0’, and *L* unknown information symbols to form a frame. There, the 2*T* symbols, which are known to the backscatter node and reader node, form the “training sequence”, as shown in Figure 12. We will discuss how to use the training sequence to detect original bits without CSI estimation in subsequent sections.

### 4.2. Power Average Detector

In the context of analog circuits [8], a “double filter” detector is proposed. The detector circuit has two lowpass filters with different cutoff frequencies. A short-term filter produces the signal envelope, and a long-term filter produces the average signal, which removes the high-frequency component of the ambient RF signal. Then, the comparator compares the two output voltage levels to identify the original information bits. In this framework, we propose using a power average detector. In digital analysis [26,29], it is common to consider a power set of the received signal in one backscatter symbol time slot. In this detector scheme, the power set offers a statistical representation of each backscatter symbol, replacing the signal envelope produced by the “short-term filter”. Representing the results as a statistical result, we have

(26) $$\left\{\begin{array}{l} V_{i}^{j}=\sum_{n=1}^{N}{\left\|y_{⊥}^{}\left[(i-1)N+n\right]\right\|}^{2}\\ H_{i}^{j}=\sum_{n=1}^{N}{\left\|y_{//}^{}\left[(i-1)N+n\right]\right\|}^{2} \end{array}\right.,\;i\in \left\{1,2,\cdots ,2T+L\right\},$$

as the power sets on the two polarization patterns of the *i*-th symbol in the *j*-th frame.

Benefiting from the training sequence and considering that the channel coefficients remain constant within each coherent frame, it is feasible to estimate the average power from the known symbols. From the training sequence setup shown in Figure 12 and the dual-polarization Manchester coding shown in Figure 10, we have the estimated average power on the two polarization patterns as

(27) $$\left\{\begin{array}{l} {\bar{V}}^{j}=\frac{1}{2T}\sum_{m=1}^{2T}\sum_{n=1}^{N}{\left\|y_{⊥}^{}\left[(m-1)N+n\right]\right\|}^{2}\\ {\bar{H}}^{j}=\frac{1}{2T}\sum_{m=1}^{2T}\sum_{n=1}^{N}{\left\|y_{//}^{}\left[(m-1)N+n\right]\right\|}^{2} \end{array}\right.,$$

which can be seen as the average signal from the “long-term filter”. Then, the “zero-mean” symbol power set can be represented as

(28) $$\left\{\begin{array}{l} {\tilde{V}}_{i}^{j}=V_{i}^{j}-{\bar{V}}^{j}\\ {\tilde{H}}_{i}^{j}=H_{i}^{j}-{\bar{H}}^{j} \end{array}\right.,\;i\in \left\{1,2,\cdots ,2T+L\right\}.$$

Hence, the different mean values of backscatter symbol power on different polarization patterns are effectively normalized. After we obtain the “zero-mean” symbol power set, the symbol power on one polarization pattern can be either larger or smaller than the symbol power on the other pattern in each symbol time slot. These relative power levels can be used to determine the two original information bits of each backscatter symbol in each frame. As the variance of backscatter symbol power still varies across polarization patterns and the ambient backscatter signal can either increase or decrease the received signal power at the reader node, it is essential to estimate the power level relationship between the two polarization patterns from the first *T* Manchester coding symbols ‘1’ in the training sequence. Define $Z_{⊥}^{j}$ and $Z_{//}^{j}$ as

(29) $$Z_{⊥}^{j}=\frac{1}{T}\sum_{u=1}^{T}{\tilde{V}}_{u}^{j},Z_{//}^{j}=\frac{1}{T}\sum_{u=1}^{T}{\tilde{H}}_{u}^{j}.$$

Then, if $Z_{⊥}^{j}>Z_{//}^{j}$, when ${\tilde{V}}_{i}^{j}>{\tilde{H}}_{i}^{j}$ then the *i*-th symbol in the *j*-th frame will be demodulated to bit ‘1’; else, it will be bit ‘0’. Otherwise, if $Z_{⊥}^{j}\leq Z_{//}^{j}$, when ${\tilde{V}}_{i}^{j}\leq {\tilde{H}}_{i}^{j}$ then the *i*-th symbol in the *j*-th frame will be demodulated to bit ‘1’; else, it will be bit ‘0’. Thus, when the “zero-mean” symbol power on different polarization patterns of an unknown data symbol bears a similar relationship to the first *T* training sequence symbols, this unknown data symbol indicates the same original information bit as the first *T* training sequence symbols.

Therefore, the power average detector can address the power imbalance problem by shifting from computing a detection threshold to finding the relationships between power symbols on different polarization patterns, regardless of which polarization pattern receives higher symbol power or whether the ambient backscatter signal increases or decreases the received symbol power in either polarization pattern.

We summarize the algorithm of the power average detector as follows (Algorithm 1):
**Algorithm 1** Power average detector**Input:** The received signal samples vectors of the *j*-th frame: $\left[y_{⊥}[1],y_{⊥}[2],\cdots ,y_{⊥}[(i-1)N+n],\cdots ,y_{⊥}[(2T+L)N]\right]$, $\left[y_{//}[1],y_{//}[2],\cdots ,y_{//}[(i-1)N+n],\cdots ,y_{//}[(2T+L)N]\right]$ **Output:** $\mathrm{The}\;\mathrm{detected}\;\mathrm{symbols}:\;\left[d[2T+1],d[2T+2],\cdots ,d[2T+L]\right]$ 1:  Training Phase: For *m* from 1 to 2*T* 2:      $\mathrm{Compute}\;V_{m}^{j}$$\;\mathrm{and}\;H_{m}^{j}$$\;\mathrm{by}\;(26),\;\mathrm{compute}\;{\bar{V}}^{j}$$\;\mathrm{and}\;{\bar{H}}^{j}$ by (27) 3:      $\mathrm{Compute}\;Z_{⊥}^{j}$$\;\mathrm{and}\;Z_{//}^{j}$ by (28) and (29) 4:  end for 5:  For *l* from 1 to *L* 6:      $\mathrm{Compute}\;{\tilde{V}}_{2T+l}^{j}$$\;\mathrm{and}\;{\tilde{H}}_{2T+l}^{j}$ by (28) 7:      $\mathrm{if}\;Z_{⊥}^{j}>Z_{//}^{j}$ 8:          $\mathrm{if}\;{\tilde{V}}_{2T+l}^{j}>{\tilde{H}}_{2T+l}^{j}$, then let d[2T+l]=1, else d[2T+l]=0, end if 9:      $\mathrm{else}\;\mathrm{if}\;{\tilde{V}}_{2T+l}^{j}\leq {\tilde{H}}_{2T+l}^{j}$, then let d[2T+l]=1, else d[2T+l]=0, end if 10:    end if 11:  end for 12:  Return $\left[d[2T+1],\;d[2T+2],\cdots ,d[2T+L]\right]$


After we obtain the detection results, we can calculate the BER and the throughput as in (20).

### 4.3. K-Means Clustering Detector

Although the power average detector is simple and intuitive, it may cause information loss because of the subtraction of the approximate average power, as outlined in (28). Detectors that can demodulate symbols directly from the original unbalanced power set on the two polarization patterns are required. From the analysis in Section 4.2 and Figure 11, we know that the most important factor in detecting the Manchester symbols in simultaneous backscatter mode is identifying the relationship between symbol powers on different polarization patterns. Therefore, an appealing approach is to directly cluster the symbols that exhibit similar relationships in their symbol power on different polarization patterns.

K-means algorithm is a kind of clustering algorithm in the expectation-maximization (EM) framework that has the advantages of computation simplicity, few parameters and good flexibility across different datasets. Combine $V_{i}^{j}$ and $H_{i}^{j}$ into a 2-dimensional vector $Z_{i}^{j}=\left\{V_{i}^{j},H_{i}^{j}\right\}$, representing the power sets on the two polarization patterns of each symbol.

In E-step, calculate

(30) $$Dist(k,l)={\left\|Z_{l}^{j}-\mu_{k}^{j}\right\|}^{2},\;k\in \left\{0,1\right\},l\in \left\{1,2,\cdots ,L\right\}$$

to represent the Euclidean distance between each power set of unknown symbol *l* in length *L* data and *K* = 2 clustering centers $\mu_{k}^{j}$. Then, label symbol *l* into class *k_l_* by choosing $k_{l}=\arg\min_{k}Dist(k,l)$.

In M-step, denote the loss function as

(31) $$J=\sum_{l=1}^{L}\sum_{k=1}^{K}r_{lk}{\left\|Z_{l}^{j}-\mu_{k}^{j}\right\|}^{2},\;r_{lk}=\left\{\begin{array}{l} 1,\;if\;k_{l}=k\\ 0,\;else \end{array}\right.$$

in order to represent the density of each clustering class, which consists of all the distances between the symbol power sets in class *k* and the clustering center $\mu_{k}^{j}$. The purpose of clustering is to merge similar samples into the same class, and it is best to minimize the loss function. In mathematical terms, this means

(32) $$\frac{\partial J}{\partial \mu_{k}^{j}}=2\sum_{l=1}^{L}r_{lk}\left(Z_{l}^{j}-\mu_{k}^{j}\right),$$

while the partial derivative represents the trend in the variation of the loss function with $\mu_{k}^{j}$.

When $\frac{\partial J}{\partial \mu_{k}^{j}}=0$, the loss function is minimized. Then, we have

(33) $$\mu_{k}^{j}{}^{\prime }=\frac{\sum_{l=1}^{L}r_{lk}Z_{l}^{j}}{\sum_{l=1}^{L}r_{lk}}$$

representing the new cluster center as the centroid of all samples in the same class. Update $\mu_{k}^{j}{}^{\prime }$ instead of $\mu_{k}^{j}$, then repeat the E-step and the M-step until the labels of all *l* symbols become constant or exceed the limit execution number.

However, as an unsupervised machine-learning algorithm, the K-means clustering algorithm also suffers from the local optimality issues caused by its sensitivity to the initial choice of cluster centers. Moreover, unsupervised machine learning groups samples into classes but does not give the meaning of each class. However, exact demodulated information bits are required in the detector scheme. In order to overcome these issues, we use the training-sequence symbols as the initial clustering centers. Note that the labels from the training-sequence symbols are not really used in the EM steps of the algorithm; they simply indicate the corresponding information bits for each output class. Thus, a training sequence of length *T* = 1 is sufficient. However, the K-means clustering detector comes with a trade-off, as it increases computation complexity and time delay. The detection output is available only after the whole frame has been processed, and the algorithm requires several iterations to converge.

Therefore, the K-means clustering detector can address the power imbalance problem. It can self-adaptively and directly cluster the backscatter symbols that exhibit similar relationships in terms of symbol power across different polarization patterns, avoiding the loss associated with estimating average power.

We summarize the algorithm used by the K-means clustering detector as follows (Algorithm 2):
**Algorithm 2** K-means clustering detector**Input:** The received signal samples vectors of the *j*-th frame: $\left[y_{⊥}[1],y_{⊥}[2],\cdots ,y_{⊥}[(i-1)N+n],\cdots ,y_{⊥}[(2+L)N]\right]$, $\left[y_{//}[1],y_{//}[2],\cdots ,y_{//}[(i-1)N+n],\cdots ,y_{//}[(2+L)N]\right]$ **Output:** The detected symbols: $\left[d[3],d[4],\cdots ,d[2+L]\right]$ 1:  For *m* from 1 to 2 + *L* 2:      $\mathrm{Compute}\;Z_{m}^{j}=\left\{V_{m}^{j},H_{m}^{j}\right\}$ by (26) 3:  end for 4:  $\mathrm{Set}\;\mathrm{initial}\;\mathrm{clustering}\;\mathrm{centers}:\;\mu_{1}^{j}=Z_{1}^{j}$$,\;\mu_{0}^{j}=Z_{2}^{j}$ 5:  $\mathrm{Set}\;\mathrm{initial}\;\mathrm{labels}:\;\left[k_{3}^{j},k_{4}^{j},\cdots ,k_{2+L}^{j}\right]=\left[0,0,\cdots ,0\right]$ 6:  For *epochs* from 1 to 1000 7:      For *l* from 1 to *L* 8:          For *k* from 0 to 1 9:              Compute Dist(k,2+l) by (30) 10:          end for 11:          $\mathrm{Choose}\;\mathrm{new}\;\mathrm{label}\;\mathrm{by}\;k_{2+l}^{j}{}^{\prime }=\arg\min_{k}Dist(k,2+l)$ 12:      end for 13:      $\mathrm{if}\;\left[k_{3}^{j}{}^{\prime },k_{4}^{j}{}^{\prime },\cdots ,k_{2+L}^{j}{}^{\prime }\right]=\left[k_{3}^{j},k_{4}^{j},\cdots ,k_{2+L}^{j}\right]$ 14:          **Break** 15:      $\mathrm{else}\;\mathrm{let}\;\left[k_{3}^{j},k_{4}^{j},\cdots ,k_{2+L}^{j}\right]=\left[k_{3}^{j}{}^{\prime },k_{4}^{j}{}^{\prime },\cdots ,k_{2+L}^{j}{}^{\prime }\right]$ 16:      end if 17:      For *k* from 0 to 1 18:          $\mathrm{Compute}\;\mathrm{new}\;\mathrm{clustering}\;\mathrm{centers}\;\mu_{k}^{j}{}^{\prime }$$\;\mathrm{by}\;(33),\;\mathrm{let}\;\mu_{k}^{j}=\mu_{k}^{j}{}^{\prime }$ 19:      end for 20:  end for 21:  $\mathrm{Check}\;\mathrm{the}\;\mathrm{training}\;\mathrm{symbol}’s\;\mathrm{label}\;\mathrm{by}\;k_{1}^{j}=\arg\min_{k}Dist(k,1)$ 22:  $\mathrm{If}\;k_{1}^{j}=1$ 23:      $\left[d[3],d[4],\cdots ,d[2+L]\right]=\left[k_{3}^{j},k_{4}^{j},\cdots ,k_{2+L}^{j}\right]$ 24:  $\mathrm{else}\;\left[d[3],d[4],\cdots ,d[2+L]\right]=\left[1-k_{3}^{j},1-k_{4}^{j},\cdots ,1-k_{2+L}^{j}\right]$ 25:  end if 26:  $\mathrm{Return}\;\left[d[3],d[4],\cdots ,d[2+L]\right]$


After we obtain the detection results, = we can calculate the BER and the throughput, as in (20).

## 5. Simulation Results

In this section, theoretical and simulation results are provided to validate the feasibility of our proposed DPAm node structures and the performance of the proposed detection methods. We simulate a typical office environment using the baseband communication model analyzed above. The dual-polarized antenna of the backscatter node is as shown in Figure 2, and the ambient RF source node operates at a center frequency of 2.4 GHz. As the default simulation settings, we consider the transmitted power of the ambient RF source with $SNR=P_{s}/N_{w}=$ 15 dB. As the physical distance between the ambient RF source node and the reader node is much greater than the distance between the backscatter node and the reader node, we set $h_{sr},h_{sb}∼CN(0,1)$, $h_{br}∼CN(0,10)$ [24,26], with reflection coefficient *η* = 0.5 and backscatter symbol data rate *R_b_* = 10^4^. Without loss of generality, we assume that *α_sr_* and *α_sb_* are uniform distributions that vary independently in each coherent frame. We first consider an ideal situation $\alpha_{sr},\alpha_{sb}∼U(40{}^{\circ},50{}^{\circ})$, which means that the polarization patterns of both the backscatter node and the reader node will receive approximately equal signal power, a common assumption in communications. In addition, we set the training sequence length to *T* = 5 for the power average detector and *T* = 1 for the K-means clustering detector. We set the data length in one frame to *L* = 50 and the backscatter symbol-sampling rate to *N* = 50. All simulation results are obtained by averaging 10^5^ Monte Carlo realizations (i.e., AmBC frames).

**Figure 13:**
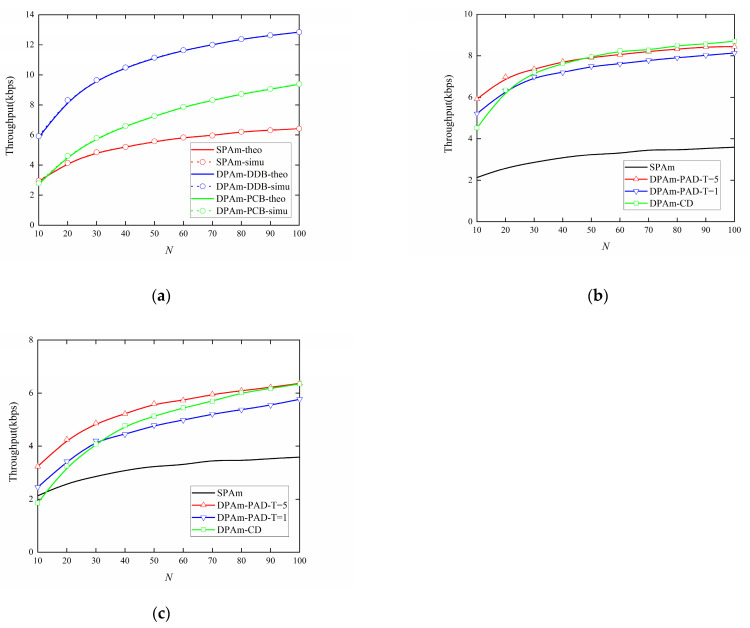
Impact of sampling rate *N* on the Throughput performance: (**a**) Parallel backscatter mode; (**b**) Simultaneous backscatter mode with DDB node structure; (**c**) Simultaneous backscatter mode with PCB node structure.

Figure 13a shows the impact of sampling rate *N* on the throughput performance of the parallel backscatter mode in the DPAm system with different backscatter node structures and the SPAm system. As can be observed, our proposed DPAm nodes significantly improve the throughput compared to the SPAm system as *N* increases. The DDB node structure can almost double the throughput compared to the SPAm system for each value of *N*. However, the PCB node structure shows less improvement in throughput, with throughput almost equal to that of the SPAm at *N* = 10, about 1.3 times better than that of the SPAm at *N* = 50, and about 1.46 times better than that of the SPAm at *N* = 100. The main reason for this phenomenon is that although the single-polarized input antenna and the Wilkinson power divider maintain fair power division between the backscatter signals on the two polarization patterns, they result in significant power loss for the backscatter signals. This power loss is also unfavorable for symbol detection and leads to a decrease in throughput performance. Moreover, the simulation results strongly corroborate the theoretical results.

Figure 13b,c also provide a comparative analysis of the impact of sampling rate *N* on the throughput performance of the simultaneous backscatter mode in the DPAm system. The comparison includes different detectors, different backscatter node structures, and the SPAm system. As *N* increases, both the node structure and the detector show better throughput performance. For each backscatter node structure, a significantly better throughput performance can be achieved by utilizing the power average detector with a longer training sequence. This improvement is caused by the fact that a longer training sequence will yield a more accurate average power estimation. It can also be readily observed that when the DDB node structure is utilized, the maximum throughput performance can be more than double the throughput performance of the SPAm system at the same sampling rate and about 2.42 times better than the throughput performance of the SPAm system at *N* = 100. When the PCB node structure is utilized, it also achieves better throughput performance than the SPAm system in most cases; its maximum throughput performance is about 1.76 times better than the throughput performance of the SPAm system at *N* = 100. These results validate that our proposed simultaneous backscatter mode is useful when the parameters are unknown. We also note that when *N* < 30, the throughput performance of the clustering detector is inferior to that of the power average detector with *T* = 1. This result suggests that the clustering detector requires more statistical received signal power to distinguish clearly among the received power sets. Conversely, when *N* > 50, the clustering detector achieves throughput performance comparable to that of the power average detector with *T* = 5. This feature is particularly beneficial for scenarios in which communication resources are limited so only a short training sequence is available. Moreover, when each backscatter node structure is utilized, the amplitude of the variation of the simultaneous backscatter mode from *N* = 10 to *N* = 100 is smaller than in the parallel backscatter mode. This result proves that the simultaneous backscatter mode with Manchester coding can improve the stability of DPAm system, which means that a lower *N* will result in less reduction in throughput performance compared to the parallel backscatter mode and may save communication resources. As the simultaneous backscatter mode does not involve estimation of CSI, it yields lower absolute values for throughput performance than the parallel backscatter mode, but it is more practical.

**Figure 14:**
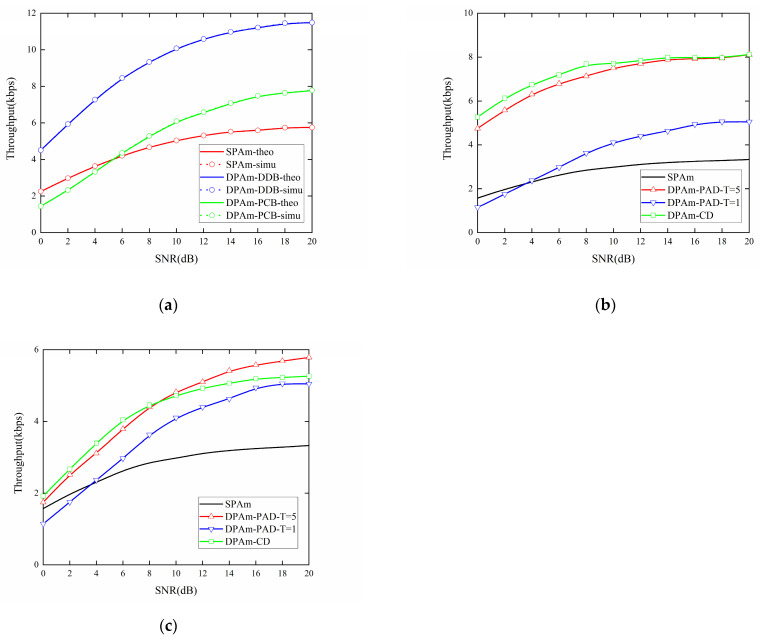
Impact of SNR on the throughput performance: (**a**) parallel backscatter mode; (**b**) simultaneous backscatter mode with DDB node structure; (**c**) simultaneous backscatter mode with PCB node structure.

Figure 14a presents a comparison of the impact of SNR on the throughput performance of the parallel backscatter mode in the DPAm system with different backscatter node structures, and on the SPAm system. The DDB node structure can also almost double the throughput compared to the SPAm system for each SNR level, and the performance of the PCB node structure is about 1.35 times better than that of the SPAm at SNR = 20 dB. However, when SNR < 6 dB, the throughput performance of the PCB node structure is worse than that of the SPAm system. The simulation results are also strongly corroborated by the theoretical results. Figure 14b,c also compare the impact of SNR on the throughput performance of the simultaneous backscatter mode in the DPAm system. The comparison includes different detectors and different backscatter node structures, as well as the SPAm system. When the DDB node structure is utilized, the maximum throughput performance can be more than double that of the SPAm system at the same SNR, which is about 2.43 times better than the throughput performance of the SPAm system at SNR = 20 dB. When the PCB node structure is utilized, it also achieves better throughput performance than SPAm system in most cases. Its maximum throughput performance is about 1.58 times better than that of the SPAm system at SNR = 20 dB. When utilizing the power average detector, the performance improvement from *T* = 1 to *T* = 5 for the DDB node structure is significantly larger than that for the PCB node structure. Moreover, when SNR < 4 dB, the throughput performance of the power average detector with *T* = 1 is even lower than that of the SPAm system. For each backscatter node structure, when SNR < 10 dB, the clustering detector has the best throughput performance. For the DDB node structure, when SNR > 10 dB, the clustering detector has throughput performance similar to that of the power average detector with *T* = 5. This result also demonstrates that the clustering detector is more flexible in low and high SNR conditions and across different backscatter node structures. Moreover, when each backscatter node structure is utilized, the amplitude of the variation in throughput performance in the simultaneous backscatter mode, from SNR = 0 dB to SNR = 20 dB, is less pronounced than in the parallel backscatter mode. This result also demonstrates that the simultaneous backscatter mode with Manchester coding could improve the stability of the DPAm system, which means that a lower SNR will result in a smaller reduction throughput performance compared to what is experienced in the parallel backscatter mode. Its performance may also suffer less in an environment with a variable SNR.

**Figure 15:**
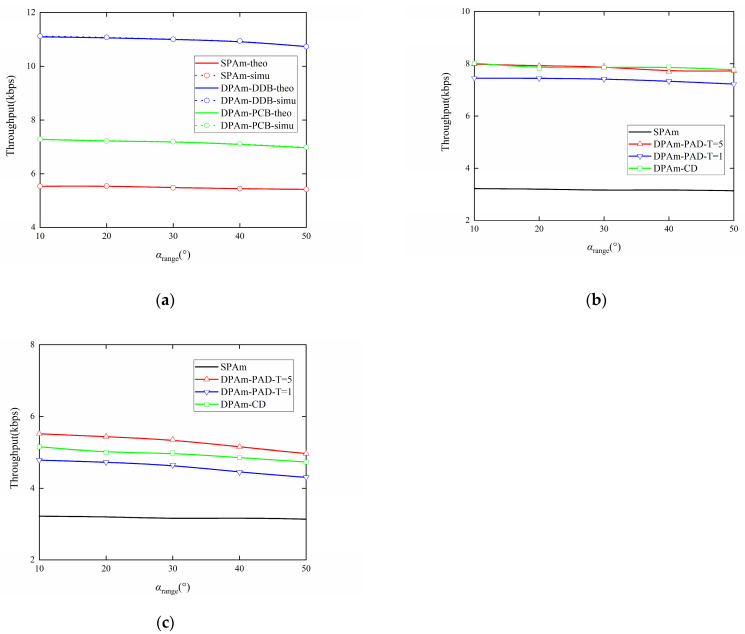
Impact of included angle changing range on the Throughput performance: (**a**) Parallel backscatter mode; (**b**) Simultaneous backscatter mode with DDB node structure; (**c**) Simultaneous backscatter mode with PCB node structure.

Figure 15a presents a comparison showing how the range of change in the included angle affects the throughput performance of the parallel backscatter mode in the DPAm system, considering different backscatter node structures, as well as the SPAm system. Figure 15b,c also present a comparison of the impact of the range of change in the included angle on the throughput performance of the simultaneous backscatter mode in the DPAm system. This comparison also considers different detectors, different backscatter node structures, and the SPAm. To further explore the effect of the included angles in the DPAm system, we set the included angle to $\alpha_{sr},\alpha_{sb}∼U(\alpha_{center}-\alpha_{range}/2,\alpha_{center}+\alpha_{range}/2)$. In the simulation settings, we fixed *α_center_* = 45°, as in the default settings, and changed *α_range_*. All throughput performance decreases as *α_range_* increases, but the size of the decrease is small. This result indicates that the range of change in the included angles does not cause strong interference in AmBC systems. For the parallel backscatter mode, the DDB node structure can also double throughput compared to the SPAm system for each *α_range_* case, while the PCB node structure achieves throughput performance just about 1.3 times better than that of the SPAm for each *α_range_* case. For the simultaneous backscatter mode, when the DDB node structure is utilized, the maximum throughput performance is about 2.5 times better than that of the SPAm for each *α_range_* case, and the clustering detector has throughput performance similar to that of the power average detector with *T* = 5. When the PCB node structure is utilized, the maximum throughput performance is about 1.6 times better than that of the SPAm for each *α_range_* case and the clustering detector still has better throughput performance than the power average detector with *T* = 1. This result also demonstrates that the clustering detector has better self-adaptivity than the power average detector in complex *α_range_* cases given the same length of training sequence.

**Figure 16:**
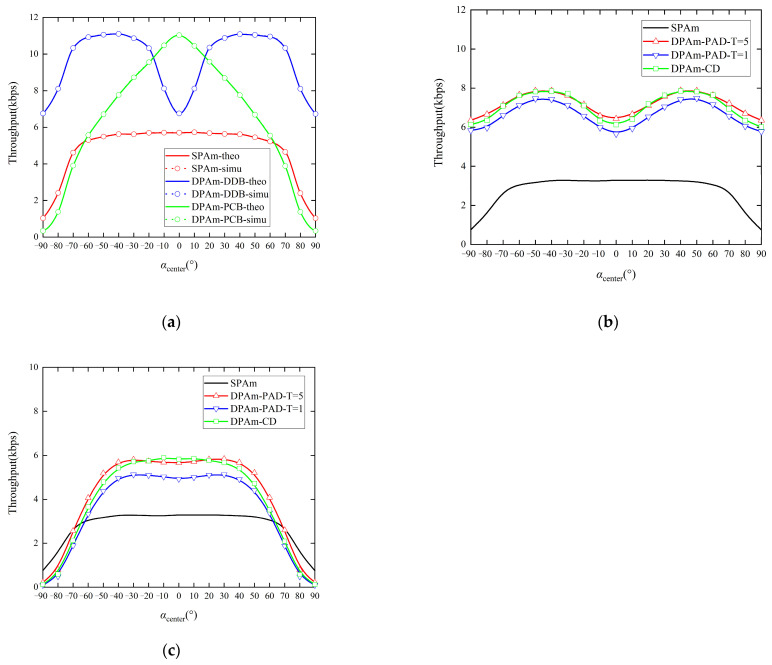
Impact of included angle center on the throughput performance: (**a**) parallel backscatter mode; (**b**) simultaneous backscatter mode with DDB node structure; (**c**) simultaneous backscatter mode with PCB node structure.

Figure 16a presents a comparison of the impact of the included angle center on the throughput performance of the parallel backscatter mode in the DPAm system. The comparison includes different backscatter node structures and the SPAm system. Figure 16b,c also present a comparison of the impact of included angle center on the throughput performance of the simultaneous backscatter mode in the DPAm system with different detectors and different backscatter node structures, and this analysis also includes the SPAm. In this simulation, we fixed *α_range_* = 20° and increased *α_center_*. Our two proposed DPAm node structures display notable differences in throughput performance depending on the center of the included angle. Specifically, the DDB node structure has better throughput performance when *α_center_* is close to ±45°, while the PCB node structure has better throughput performance when *α_center_* is close to 0°. This phenomenon can be intuitively understood considering that the PCB node structure depends mainly on the received power at the input antenna, when *α_center_* are closer to 0°, the input antenna receives more power to backscatter symbols that is beneficial to demodulation. In contrast, the DDB node structure can utilize the received power on both polarization patterns simultaneously, so it is less sensitive to *α_center_* than the PCB node structure. When *α_center_* is close to ±45°, the two polarization patterns of the DDB node receive similar levels of signal power, enabling a more balanced backscatter of information and symbol detection and thus the best throughput performance. In addition, when the DDB node structure is utilized in each backscatter mode, the throughput performance is significantly better than that of the SPAm system, while when the PCB node structure is utilized in each backscatter mode, the throughput performance is better than that of the SPAm system only when $\alpha_{center}\in [-60{}^{\circ},60{}^{\circ}]$. When *α_center_* is close to ±90°, the input antenna of the PCB node structure receives little power. This situation is unfavorable for backscatter symbols and leads to worse throughput performance. Moreover, the clustering detector for the simultaneous backscatter mode has throughput performance similar to that of the power average detector with *T* = 5 when *α_center_* is changing. Considering the real-world scenarios in which the included angles are difficult to measure, the robustness of the clustering detector is more relevant. It performs well with short training sequences and in complex cases involving changes in the included angles. We also note that the amplitude of the variation in the simultaneous backscatter mode from *α_center_* = −90° to *α_center_* = 90° is smaller than that observed in the parallel backscatter mode. This result also proves that use of the simultaneous backscatter mode with Manchester coding could improve the stability of the DPAm system, which means that a changing *α_center_* will yield result in less pronounced deterioration in throughput performance than would occur in the parallel backscatter mode. Additionally, the simultaneous mode is better suited to handling complex environments in which the included angles are changing.

**Figure 17:**
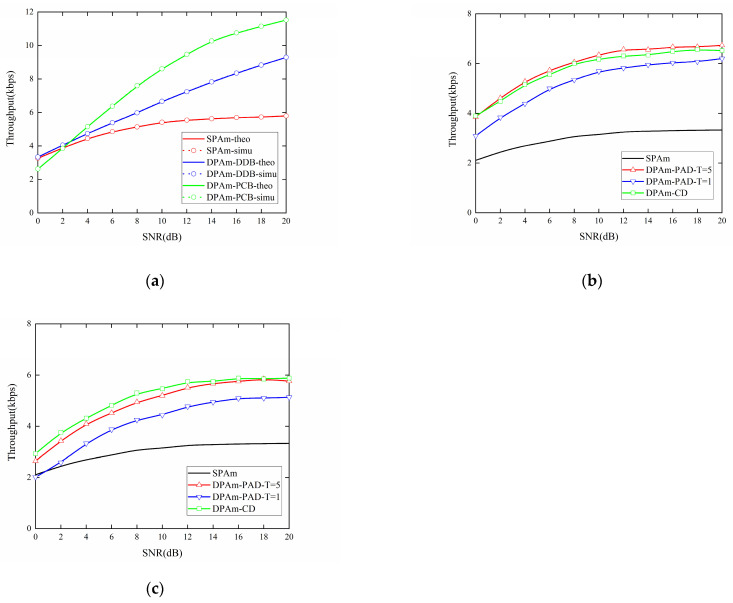
Throughput performance with $\alpha_{sr},\alpha_{sb}∼U(-20{}^{\circ},20{}^{\circ})$: (**a**) parallel backscatter mode; (**b**) simultaneous backscatter mode with DDB node structure; (**c**) simultaneous backscatter mode with PCB node structure.

In addition, we consider a specific case with $\alpha_{sr},\alpha_{sb}∼U(-20{}^{\circ},20{}^{\circ})$, indicating that the polarization of the transmit antenna and the backscatter antenna and the reader antennas are well (but not perfect) aligned. Figure 17a illustrates the throughput performance of the parallel backscatter mode in the DPAm system, comparing different backscatter node structures and the SPAm system, and Figure 17b,c also illustrate the throughput performance of the simultaneous backscatter mode in the DPAm system. These figures include comparisons across various detectors and backscatter node structures, as well as the SPAm system. In the parallel backscatter mode alone, the PCB node structure has the best throughput performance at SNR > 4 dB. Under those conditions, the maximum throughput performance is about 1.98 times better than that of the the SPAm system at SNR = 20 dB, while in all other cases, the throughput performance of the PCB node structure is inferior to that of the DDB node structure. The main reason for this difference is analyzed above: although the single-polarized input antenna and the Wilkinson power divider maintain the fairness in distributing power between the two polarization patterns, they experience significant power loss for backscatter signals. In turn, this loss negatively affects symbol detection and reduces throughput performance. The input antenna can maximize the ambient RF signal power for backscatter only when the included angles are close to 0°. Although the PCB node structure is designed mainly to address the problem of the unknown arrival direction of the ambient RF signal, the PCB node structure still must be carefully designed to maintain the performance and integrity of backscatter.

## 6. Conclusions

To extend the AmBC system into the realm of polarization diversity, in this paper, we propose direct dual-polarization and polarization-conversion backscatter node structures. In addition, we consider a parallel backscatter mode with differential coding and a simultaneous backscatter mode with Manchester coding, along with their corresponding detectors. For the parallel backscatter mode, we give analytical detection thresholds and theoretical throughput calculations. For the simultaneous backscatter mode, we propose practical detectors that are computationally simple and do not require any CSI estimation. The purpose of that model is to address the power imbalance problem in the DPAm system. Our simulation results demonstrated the feasibility and efficiency of our proposed DPAm node structures and corresponding detectors. These structures also show significantly improved throughput compared to the SPAm system. Moreover, our proposed clustering detector is more robust to short training sequences and scenarios involving complex included angles. While this paper focuses mainly on proposing a framework for a DPAm system, we also show as an example a dual-polarized smartwatch antenna to demonstrate the potential applications of these systems in wearable sensing. Such devices are also suitable for object localization and environment monitoring. To further improve the performance of the DPAm system, our future work will focus on more practical cases, including an investigation of the mutual interference between the two polarization patterns, improvements to the backscatter communication range, and incorporation of the dual-band characteristic of the antenna. We hope that this paper will inspire further research in dual polarization and that more AmBC designs that maximize the efficiency of use of communication resources will improve the performance of battery-free communications in the IoT.

## Figures and Tables

**Table 1:** Values of geometric parameters (unit: millimeters) [35].

**R1**	**R2**	**R3**	**R4**	**L1**	**W1**	**W2**
16.8	14	13	11.2	10	1.5	1
**W3**	**H1**	**H2**	**Rvia1 ^1^**	**Rvia2**	**Rvia3**	**Rvia4**
5	2.8	4	0.6	0.6	0.3	0.5

^1^ “Rvia1/2/3/4” represent the radius of the via1/2/3/4 marked in Figure 2b.

## Data Availability

Data are contained within the article.
